# Heterogeneity in Genetic Admixture across Different Regions of Argentina

**DOI:** 10.1371/journal.pone.0034695

**Published:** 2012-04-10

**Authors:** Sergio Avena, Marc Via, Elad Ziv, Eliseo J. Pérez-Stable, Christopher R. Gignoux, Cristina Dejean, Scott Huntsman, Gabriela Torres-Mejía, Julie Dutil, Jaime L. Matta, Kenneth Beckman, Esteban González Burchard, María Laura Parolin, Alicia Goicoechea, Noemí Acreche, Mariel Boquet, María Del Carmen Ríos Part, Vanesa Fernández, Jorge Rey, Mariana C. Stern, Raúl F. Carnese, Laura Fejerman

**Affiliations:** 1 Departamento de Antropología, University of Buenos Aires, Buenos Aires, Argentina; 2 Centro de Estudios Biomédicos, Biotecnológicos, Ambientales y Diagnósticos, Universidad Maimónides, Buenos Aires, Provincia de Buenos Aires, Argentina; 3 Consejo Nacional de Investigaciones Cientificas y Tecnicas, Buenos Aires, Argentina; 4 Division of Pulmonary and Critical Care, University of California San Francisco, San Francisco, California, United States of America; 5 Unit of Anthropology, Department of Animal Biology, Universitat de Barcelona, Barcelona, Spain; 6 Division of General Internal Medicine, University of California San Francisco, San Francisco, California, United States of America; 7 Institute for Human Genetics, University of California San Francisco, San Francisco, California, United States of America; 8 Medical Effectiveness Research Center for Diverse Populations, University of California San Francisco, San Francisco, California, United States of America; 9 Helen Diller Family Comprehensive Cancer Center, University of California San Francisco, San Francisco, California, United States of America; 10 Department of Epidemiology and Biostatistics, University of California San Francisco, San Francisco, California, United States of America; 11 Centro de Investigación en Salud Poblacional, Instituto Nacional de Salud Pública, Cuernavaca, Morelos, Mexico; 12 Department of Biochemistry, Ponce School of Medicine and Health Sciences, Ponce, Puerto Rico; 13 Department of Physiology and Pharmacology, Ponce School of Medicine and Health Sciences, Ponce, Puerto Rico; 14 Deptartment of Genetics, Cell Biology and Developmental Biology, University of Minnesota, Minneapolis, Minnesota, United States of America; 15 Department of Bioengineering and Therapeutic Sciences, University of California San Francisco, San Francisco, California, United States of America; 16 Facultad de Ciencias Naturales, Universidad Nacional de Salta, Salta, Argentina; 17 Facultad de Ciencias Naturales, Universidad Nacional de la Patagonia, Esquel, Chubut, Argentina; 18 Banco de Sangre del Hospital Regional de Comodoro Rivadavia, Comodoro Rivadavia, Chubut, Argentina; 19 Servicio de Hemoterapia Hospital Penna de Bahía Blanca, Bahía Blanca, Buenos Aires, Argentina; 20 Servicio de Hemoterapia Hospital de Clínicas, Universidad de Buenos Aires, Buenos Aires, Argentina; 21 Norris Comprehensive Cancer Center, University of Southern California, Los Angeles, California, United States of America; University of Cambridge, United Kingdom

## Abstract

The population of Argentina is the result of the intermixing between several groups, including Indigenous American, European and African populations. Despite the commonly held idea that the population of Argentina is of mostly European origin, multiple studies have shown that this process of admixture had an impact in the entire Argentine population. In the present study we characterized the distribution of Indigenous American, European and African ancestry among individuals from different regions of Argentina and evaluated the level of discrepancy between self-reported grandparental origin and genetic ancestry estimates. A set of 99 autosomal ancestry informative markers (AIMs) was genotyped in a sample of 441 Argentine individuals to estimate genetic ancestry. We used non-parametric tests to evaluate statistical significance. The average ancestry for the Argentine sample overall was 65% European (95%CI: 63–68%), 31% Indigenous American (28–33%) and 4% African (3–4%). We observed statistically significant differences in European ancestry across Argentine regions [Buenos Aires province (BA) 76%, 95%CI: 73–79%; Northeast (NEA) 54%, 95%CI: 49–58%; Northwest (NWA) 33%, 95%CI: 21–41%; South 54%, 95%CI: 49–59%; p<0.0001] as well as between the capital and immediate suburbs of Buenos Aires city compared to more distant suburbs [80% (95%CI: 75–86%) versus 68% (95%CI: 58–77%), p = 0.01]. European ancestry among individuals that declared all grandparents born in Europe was 91% (95%CI: 88–94%) compared to 54% (95%CI: 51–57%) among those with no European grandparents (p<0.001). Our results demonstrate the range of variation in genetic ancestry among Argentine individuals from different regions in the country, highlighting the importance of taking this variation into account in genetic association and admixture mapping studies in this population.

## Introduction

The current population of Argentina is the result of generations of intermixing between various groups, including Indigenous Americans who originally resided in this part of South America, Spanish conquistadores and Africans brought as slaves starting in the early and late 1500s respectively, and a large European immigrant population that arrived between 1870 and 1950 [1]. This process was sex-biased, frequently involving Indigenous American women and European men, as evidenced by results from mitochondrial DNA and Y-chromosome analysis [2], [3]. Further sources of admixture in the Argentina population have been introduced by local migration from the rural areas to the cities (1930–1980), and more recently, by immigration from other South American countries such as Paraguay, Peru and Bolivia (National Institute of Statistics and Census of Argentina (INDEC), 2008).

In spite of this rich history of immigration and admixture, most of the Argentine population self-identifies as of European-descent, with only 1% of the total population self-identifying as descendants of an indigenous group (INDEC, 2006). In contrast to this perception, it has been reported that a considerable proportion of the Argentine population has at least one Indigenous American ancestor [2]. Most of the studies that evaluated the distribution of genetic ancestry in Argentina included samples from the Buenos Aires province, where a great proportion of the population resides [1], [4], [5], [6]. The ancestry proportion estimates for this region by these studies ranged between 78–90% European, 15–19% Indigenous American and 2–4% African. A report on genome admixture proportions among Latin American Mestizos that included a small set of individuals from three provinces in the Argentine Northwest, Tucuman, Catamarca and Salta, reported ancestry estimates of 30%, 42% and 72% Indigenous American ancestry, respectively [7]. Finally, a study of Indigenous American genetic ancestry distribution among men from the Argentine Northeast (n = 61), the Central region (n = 153) and the Southern region (n = 32) reported estimates of 17%, 15% and 28% respectively [2]. Therefore, altogether these studies support the notion that the complex pattern of immigration and admixture in Argentina has left an imprint in the genetic composition of this country. However, large comprehensive studies across Argentina's many regions in order to characterize the genetic admixture have been lacking.

In this study we investigated the distribution of genetic ancestry in four regions of Argentina in a relatively large number of individuals (n = 441), taking into account information on the origin of each individual's grandparents. The latter allowed us to evaluate the level of concordance between grandparental origin and genetic ancestry estimates. We also compared the distribution of individual ancestry in Buenos Aires city (N = 168), the largest urban area in Argentina, to those of two other large urban areas in Latin America: Mexico City (N = 502) and San Juan de Puerto Rico (N = 133) to contextualize the observed level of variation in individual ancestry proportions of Buenos Aires with those observed in other major Latin American cities.

## Materials and Methods

### Ethics Statement

All participants provided written informed consent. The study was approved by the Human Research Protection Program, Committee of Human Research of the University of California, San Francisco, the Ethics Committee of the Hospital Italiano of Buenos Aires and the Ponce School of Medicine & Health Sciences Institutional Review Board. The Argentine Ministry of Health approved the study and the shipment of samples from Argentina to UCSF for analysis.

### Subject ascertainment

**Figure 1:**
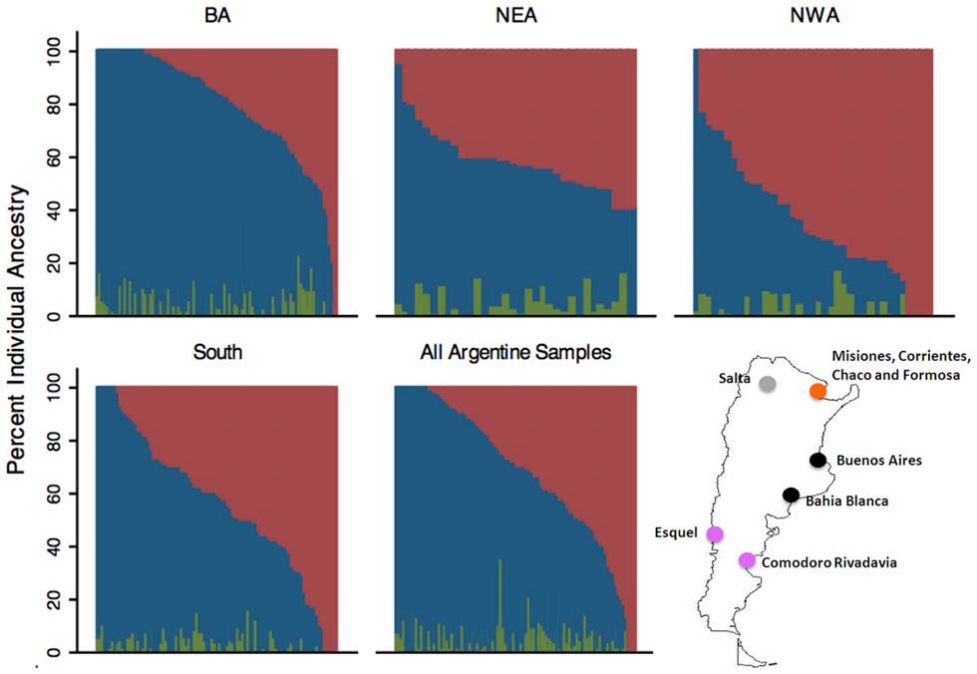
Distribution of genetic ancestry among 441 individuals from Argentina by four major regions. Each individual is represented by a vertical bar on the X-axis. Bars are divided into percent European (blue), Indigenous American (red) and African ancestry (green). BA = Buenos Aires province; NEA = Northeast; NWA = Northwest; South = South. Individuals on the X-axis are sorted based on increasing Indigenous American ancestry. On the lower right corner we include a map of Argentina indicating the location of the samples. For analysis we grouped samples by region: black: BA, pink: South, grey: NWA, orange: NEA. Samples of individuals from the NEA region (orange) were obtained from the hospitals in Buenos Aires.

Argentine men and women were randomly identified between the years 2000 and 2010 from blood donor banks within major hospitals in different regions of the country and invited to participate. All individuals were asked to donate a sample of peripheral blood, and were asked to provide information about the region/country of birth of all grandparents (Table S1). Therefore, due to the type of ascertainment used, these individuals are not expected to be fully representative of the entire region from which they were obtained. Individuals were sampled from four major regions in Argentina (n = 558): 276 individuals from the Buenos Aires province (BA) [173 individuals from the Italiano Hospital, which is private, and from the Clínicas Hospital, which is public, in the city of Buenos Aires; and 103 individuals from the Penna Hospital in Bahía Blanca]; 117 individuals from the Southern region (South) (66 from the Regional Hospital in Comodoro Rivadavia and 51 from the Zonal Hospital in Esquel); 94 individuals from the Northwest (NWA) (Centro Privado de Hemoterapia of Salta); and 71 individuals from the Northeast of the country (NEA) (Corrientes, Formosa, Chaco and Misiones provinces) who were recruited in Buenos Aires (Figure 1).

Data on genetic ancestry from Mexico City was obtained from 502 healthy Mexican women enrolled in a breast cancer case-control study [8], [9]. They were ascertained using a probabilistic multi-stage sampling design with the aim of selecting samples that were representative of the population that attended the health centers from which cases were recruited [8], [9]. Data on genetic ancestry from Puerto Rico were obtained from 141 healthy controls that participated in an ongoing private-practice-based breast cancer case-control study [10]. The women included in the present study were from the San Juan de Puerto Rico metropolitan area.

### Genotyping

A set of 106 single nucleotide polymorphisms (SNPs) that can discriminate Indigenous American, African, and European ancestry was used to estimate the proportion of genetic ancestry in individuals from Argentina, Mexico City and San Juan de Puerto Rico. Simulation studies have shown that 100 ancestry informative markers (AIMs) with allele frequency differences similar to the ones we used here are required to achieve a correlation higher than >0.9 with true ancestry [11]. AIMs used in this study were biallelic SNPs selected from the Affymetrix 100 K SNP array (Affymetrix, Santa Clara, CA) [12]. The SNPs chosen maximize information for more than one ancestral population pairing, with a large difference in allele frequency between two ancestral populations (>0.5). The ancestry informative markers are widely spaced throughout the genome and have a well-balanced distribution across all 22 autosomal chromosomes. The average distance between markers is about 24 Mb. The parental population samples that were genotyped on the Affymetrix 100 K SNP array included 42 Europeans (Coriell's North American Caucasian panel), 37 West Africans (non-admixed Africans living in London, United Kingdom, and South Carolina), and 30 indigenous Americans (15 Mayans and 15 Nahuas) [12], [13]. Genotyping of the 106 ancestry informative markers for all samples was done by Dr. Kenneth Beckman at the Biomedical Genomics Center, University of Minnesota, using a multiplex PCR coupled with single base extension methodology with allele calls using a Sequenom analyzer. Details about the 106 AIM selection, primers and reaction conditions have been previously described [12], [14], [15], [16], [17], [18], [19]. A description of the genomic location and ancestral allele frequencies for each of the AIMs is presented in Table S2.

Six of the 106 AIMs were excluded from the analysis because they had a call rate lower than 90%. Even though AIMs are expected to violate Hardy-Weinberg equilibrium more than other markers, we excluded an additional SNP due to its deviation from expected frequencies under equilibrium (p<0.0005). The final analysis included a total of 99 AIMs. We genotyped 558 individuals from Argentina, 502 from Mexico and 141 from Puerto Rico. We found complete concordance among 10 genotyped duplicates. We excluded individuals with a genotype call rate of <70% (117 from Argentina, and 8 from Puerto Rico). The final analysis included 441 samples from Argentina, 502 from Mexico and 133 from Puerto Rico. The data used in the present study is available from the authors upon request.

As part of a different ongoing study, fifty-four out of the 441 individuals were also genotyped with an Affymetrix 250 K StyI array (∼238,000 SNPs). We excluded SNPs with more than 5% of missing data and a hardy-Weinberg equilibrium p<0.00005. Since the model in ADMIXTURE [20] does not explicitly take linkage disequilibrium (LD) into consideration, LD-based SNP pruning was performed using a sliding window of 25 SNP, shifting 3 SNPs and implementing a pairwise r2 threshold of 0.8 at each step. After quality control, 118,192 SNPs remained with a genotyping rate 99.2% to use for ancestry estimation. We used this opportunity to test the genotype concordance across laboratories and platforms as well as to compare the individual ancestry estimations obtained with the two platforms (the 99 AIMs vs. 118,192 SNPs). The genotypes had a concordance rate of 98.3%.

### Statistical analysis

Individual genetic ancestry was estimated using a maximum likelihood (ML) approach [21], [22] implemented in a Java script that is available upon request from the authors. The ML model infers each individual's ancestry as a function of the probability of the genotypes observed at each locus based on the ancestral allele frequencies. The implemented likelihood method produces estimates that are highly concordant with those produced using two other available programs for individual ancestry estimation: FRAPPE [23] and STRUCTURE [24]. For the 54 individuals with genome wide data available we estimated individual ancestry using the program ADMIXTURE [20] in order to compare the results with those obtained using the 99 AIMs. ADMIXTURE is a fast maximum likelihood based method similar to FRAPPE for individual ancestry estimation that is tractable for large SNP datasets. Ancestral European, African and Indigenous American genotypes were included in the run [Africans: 58 Yorubas from HapMap (Affymetrix 6.0 platform); Europeans: 50 Spaniards and 50 Germans from POPRES [25] (Affymetrix 500 K platform); Indigenous Americans: 14 Nahuas, 21 Mayas, 24 Quechuas, and 24 Aymaras (Affymetrix 500 K platform) [26]].

Multidimensional Scaling with pairwise allele sharing distances as implemented in the program PLINK [27] was used to estimate the first and second dimensions of variation among the Buenos Aires, Mexico City and San Juan samples. The significance of the difference in the distribution of the first two dimensions between the three population groups was evaluated using the Kruskal-Wallis equality of populations rank test.

The difference in mean European/Indigenous American ancestry between the different Argentine regions was tested using the two-sample Kolmogorov-Smirnov test for equality of distribution functions. The significance of the difference in mean Indigenous American/European ancestry between the five categories defined by the presence of 0, 1, 2, 3, or 4 grandparents that reside in a particular region of Argentina was evaluated with the Kruskal-Wallis test. Non-parametric approaches were selected because the distribution of genetic ancestry deviated from normality (Shapiro-Wilk test, p<0.05). Both analyses were conducted with the program STATA 11 [28]. We also used this program to evaluate the correlation between the ancestry estimates obtained using genome wide data and those obtained using 99 AIMS.

## Results

### Individual genetic ancestry in Argentina

**Figure 2:**
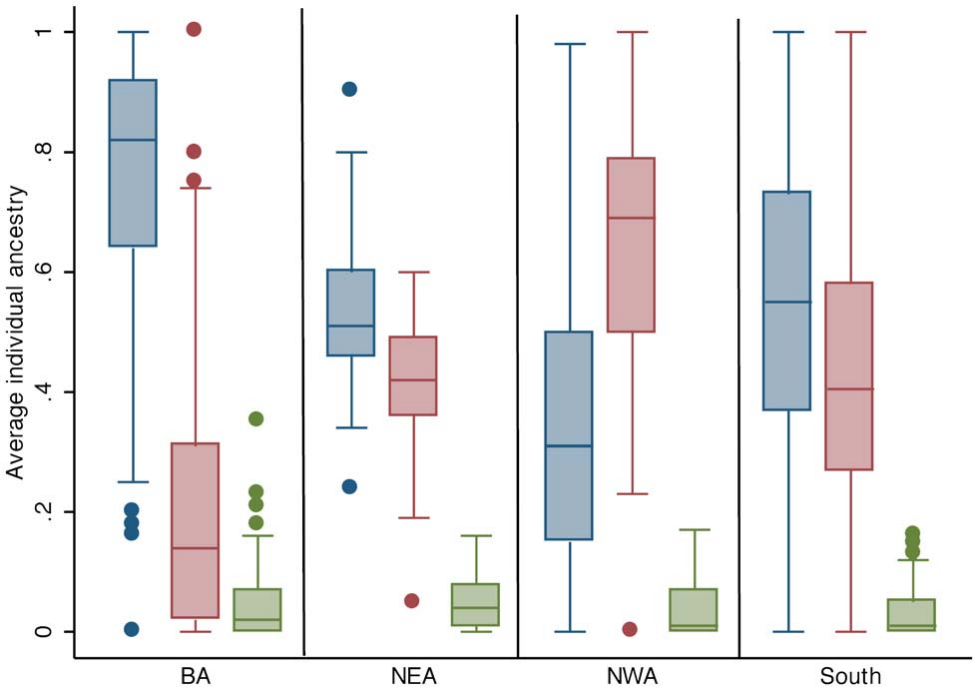
Box plots of average individual ancestry by four major Argentine regions. The blue boxes represent the European component, the red boxes the Indigenous American component and the green boxes the African component. BA = Buenos Aires province; NEA = Northeast; NWA = Northwest; South = South.

The distribution of genetic ancestry among the 441 Argentine individuals included in our study varied from 0 to 100% Indigenous American, 0 to 100% European, and 0 to 35% African ancestry (Figure 1). The average ancestry in this dataset was 65% European (95%CI: 63–68%), 31% Indigenous American (95%CI: 28–33%) and 4% African (95%CI: 3–4%). When we grouped individuals into the four major geographical regions of origin we observed different distributions (Figure 2). The mean European ancestry for BA was 76% (95%CI: 73–79%), for NWA was 33% (95%CI: 21–41), for NEA 54% (95%CI: 49–58%) and for the South 54% (95%CI: 49–59%). These observed differences in estimated proportions of European and Indigenous American ancestry across regions were statistically significant (p = 0.0001). In pairwise comparisons, individuals from NWA had significantly more Indigenous American and significantly less European ancestry compared to individuals from BA, NEA or South (p<0.0001). There were no significant differences in ancestry between NEA and South. The proportion of African ancestry was not significantly different in any of the comparisons.

### Genetic structure within the city of Buenos Aires

Individuals from Buenos Aires city were ascertained from two large hospitals, one private (n = 79) and one public (n = 89). Individuals from the private hospital had more European ancestry (80%; 95%CI: 76–85%) compared to individuals ascertained from the public hospital (76%; 95%CI: 72–80%), p = 0.028. To investigate the potential cause for this heterogeneity within the city of Buenos Aires, we determined the association between place of residence and genetic ancestry. Individuals recruited in these hospitals were residents of either the city of Buenos Aires proper, or the immediate surrounding urban areas (urban belts 1 and 2, Figure S1). Even though there was high variance of individual European and Indigenous American ancestry within every urban belt and within the city of Buenos Aires proper (Figure S2), we observed statistically significant differences in the average estimate of European or Indigenous American ancestry across these three regions, in particular, when we compared individuals from the city of Buenos Aires proper and the 1^st^ urban belt to individuals from the 2^nd^ urban belt (p = 0.01) (Table 1). Moreover, the differences in genetic ancestry estimates between the two hospitals in the city of Buenos Aires can be completely explained by the higher proportion of 2^nd^ urban belt residents in the public hospital (19% in the public vs. 11% in the private). When 2^nd^ urban belt residents were removed from the analysis, there were no significant differences in European ancestry between the two hospitals.

**Table 1:** Average individual ancestry for Buenos Aires metropolitan area, Mean (95% CI).

	European	P*	Indigenous American	P*	African	P*
All individuals combined						
Buenos Aire City (n = 98)	79 (76–83)	0.006	17 (13–20)	0.006	4 (3–5)	0.431
1^st^ uban belt (n = 47)	80 (75–86)		16 (11–22)		3 (2–5)	
2^nd^ urban belt (n = 22)	68 (58–77)		29 (20–38)		3 (1–6)	
Italian hospital (private)						
Buenos Aires City (n = 48)	84 (79–89)	0.729	11 (7–16)	0.602	5 (3–6)	0.784
1^st^ uban belt (n = 22)	75 (64–86)		21 (10–31)		4 (2–6)	
2^nd^ urban belt (n = 7)	73 (52–94)		20 (3–38)		6 (1–12)	
Clinicas' hospital (public)						
Buenos Aires City (n = 45)	77 (71–82)	0.015	20 (15–25)	0.015	3 (2–4)	0.303
1^st^ urban belt (n = 25)	85 (79–91)		12 (6–18)		2 (1–4)	
2^nd^ urban belt (n = 15)	65 (53–77)		33 (22–44)		2 (0–4)	

* p value for the two-sample Kolmogorov-Smirnov test comparing the 2^nd^ urban belt to a group that includes the Capital and the 1^st^ urban belt.

### Origin of grandparents and estimated genetic ancestry

We collected information about the region/country of birth of each individual's grandparents and we compared the mean estimated proportion of European, African and Indigenous American genetic ancestry between individuals who had 0 to 4 grandparents having been born in a particular region of Argentina, in any other Latin American country or in Europe (Table 2). As expected, the number of grandparents from Europe was a strong predictor of European ancestry as estimated by genetic markers. The average European ancestry among individuals with all 4 grandparents from Europe was 91% (88–94%) compared to 54% (51–57%) among those with 0 grandparents born in Europe. A strong correlation was also observed between the number of grandparents born in a certain region of Argentina and the average European and Indigenous American ancestries for all Argentine regions. Specifically, Indigenous American ancestry increased and European ancestry decreased with increasing number of grandparents from the NWA, NEA, South regions, and other South American (SA) countries. For example, the average European ancestry for those individuals with 4 grandparents born in the NWA was 35%; in contrast, the corresponding average European ancestry among individuals with 0 grandparents born in the NWA was 69%. This reflects the higher prevalence of Indigenous American ancestry in the NWA of Argentina. The individuals with 4 grandparents from the NWA included 12 individuals from NWA and 12 individuals from other regions of Argentina; therefore, these observed correlations are consistent even for individuals born outside the NWA region but that have NWA ancestors.

**Table 2:** Average percent ancestry (SD) by number of grandparents born in major regions of Argentina, Europe or other Latin American countries.

Region	Ancestry	0	N	1	N	2	N	3	N	4	N	p*
Europe	African	4 (5)	271	4 (4)	59	3 (4)	49	3 (4)	22	3 (3)	40	<0.001
	European	54 (25)		80 (15)		79 (20)		86 (13)		91 (9)		
	Indigenous	42 (25)		16 (15)		18 (20)		11 (13)		5 (8)		
AMBA	African	4 (5)	356	3 (5)	23	3 (4)	27	3 (4)	24	5 (5)	11	<0.001
	European	61 (26)		78 (19)		87 (13)		89 (10)		83 (13)		
	Indigenous	35 (26)		19 (18)		10 (13)		8 (11)		12 (12)		
Center	African	3 (4)	324	3 (3)	33	6 (8)	40	3 (5)	21	3 (4)	23	<0.001
	European	61 (28)		80 (16)		75 (18)		80 (15)		76 (16)		
	Indigenous	35 (28)		17 (16)		19 (16)		17 (16)		21 (15)		
NWE	African	4 (5)	372	5 (7)	8	3 (4)	22	5 (5)	15	4 (5)	24	<0.001
	European	69 (24)		68 (18)		51 (25)		26 (20)		35 (17)		
	Indigenous	27 (24)		27 (15)		46 (25)		69 (22)		61 (18)		
NEA	African	3 (4)	396	3 (3)	7	8 (8)	12	4 (5)	12	6 (4)	14	0.003
	European	66 (27)		81 (12)		55 (18)		59 (10)		49 (14)		
	Indigenous	30 (27)		16 (13)		37 (13)		37 (9)		44 (13)		
South	African	4 (5)	386	4 (6)	6	3 (3)	21	2 (2)	6	1 (2)	22	<0.001
	European	68 (25)		64 (12)		59 (23)		44 (29)		34 (26)		
	Indigenous	28 (25)		32 (15)		38 (23)		54 (29)		65 (27)		
Center-west	African	4 (5)	416	5 (7)	7	3 (3)	10	0 (0)	1	3 (4)	7	0.651
	European	65 (27)		70 (22)		71 (20)		25 (0)		66 (18)		
	Indigenous	31 (26)		25 (19)		26 (20)		75 (0)		31 (17)		
South America	African	4 (5)	336	3 (4)	20	5 (5)	31	3 (4)	8	3 (4)	46	<0.001
	European	69 (26)		63 (29)		51 (24)		63 (16)		47 (18)		
	Indigenous	27 (26)		34 (29)		44 (24)		34 (18)		50 (19)		

AMBA = Buenos Aires Metropolitan Area.

NWE = Northwest.

NEA = Northeast.

South America = Origin from other South American Countries.

* P value for Kruskal-Wallis equality of populations rank test evaluating the significance of the difference in mean Indigenous American/European ancestry between the 0 to 4 origin of grandparent categories for each region.

### Comparison of AIMs and genome wide data

In an effort to validate the ancestry estimates obtained with our set of AIMs, we next compared individual genetic ancestry estimates obtained with the set of 99 AIMs to those obtained with a set of 118,192 SNPs in a group of 54 individuals within our Buenos Aires sample. The correlation coefficients for the European and Indigenous American estimates were 0.90 and 0.93 respectively (Table S3). African ancestry showed a small level of correlation (correlation coefficient 0.12).

### Comparison of genetic ancestry in Buenos Aires City, Mexico City and San Juan de Puerto Rico

We projected the 168 samples from Buenos Aires City, 502 samples from Mexico City, 133 Puerto Rican samples from San Juan and 109 ancestral individuals (Indigenous Americans, Africans and Europeans) within the same space defined by the 1^st^ and 2^nd^ dimensions of a multidimensional scaling analysis (Figure S3). Whereas the three populations showed a similar degree of dispersion between individuals important differences in average ancestry were observed across the three cities: Buenos Aires 79%, 17%, 4%; Mexico City 28%, 68%, 4%; San Juan 70%, 11%, 19%, for European, Indigenous American and African ancestry, respectively (p = 0.0001 for a Kruskal-Wallis rank test).

## Discussion

We investigated the individual genetic ancestry proportions among individuals from four regions in Argentina and demonstrated their variation across and within regions, with the NWA region having the most striking difference in European and Indigenous American ancestry proportions compared to all other regions. Moreover, we found that within Buenos Aires City there were modest but statistically significant differences in genetic ancestry across different urban regions. In this respect, our results add to the previously published descriptions of genetic ancestry distribution in Argentina by providing more extensive sampling and genetic ancestry estimates that are based on a relatively large set of AIMs.

The genetic diversity of various Latin American populations has been previously described [29], [30], [31], [32], [33], [34], [35], [36], [37], [38]. A study in thirteen Mestizo populations from seven countries has shown differential ancestral contribution patterns between and within groups [7]. Similarly, a study in Puerto Rico showed regional differences in the distribution of individual genetic ancestry within the country [19]. The authors cautioned that in admixture mapping studies conducted in Puerto Rico the statistical power of the study and the possibility of confounding by admixture, would be influenced by the region of origin of the individuals.

There have been previous studies that focused on the genetic admixture characteristics of the Argentine population. Our group has previously investigated the proportion of African ancestry in individuals from Buenos Aires City [5] and reported that although the average proportion of African ancestry was low (2.2%), approximately 10% of the individuals in the study accounted for it. Seldin *et al.* [39] genotyped a set of 78 AIMs in 94 Argentine individuals and found a similar level of African admixture. Importantly, a very large variance was observed in the individual Indigenous American contribution that ranged from 1.5% to 84.5%. The individuals were ascertained from five different Argentine cities within the central region of the country. Martínez Marignac *et al* [18] investigated the distribution of genetic ancestry among 87 individuals from La Plata, Province of Buenos Aires using a set of 5 AIMs. They reported that European ancestry varied between 48% and 75%, Indigenous American ancestry between 20% to 45% and that average African ancestry was about 5%. These results suggest a widespread mixing process given that all 87 individuals had contributions from the three parental populations, which differs strongly from our results. Our estimates of individual European or Indigenous American ancestry in Buenos Aires range from 0 to 100, which supports a more restricted mixing process. Our estimates are consistent with the genealogical data we obtained for each individual, and with the historical information that indicates that most European immigration occurred 3 or 4 generations ago. One possible explanation for the discrepancy between our results and those of Martinez Marignac *et al.* [6] is the different origin of the ascertained individuals. Another possibility is error in the estimates of individual genetic ancestry due to the reduced number of AIMs used in Martinez Marignac's study.

A more recent study by Corach *et al* [2] used a regional approach including individuals from South, Central and NEA regions and reported significant differences between them [2]. Specifically, they reported higher Indigenous American ancestry among individuals from South (28%), relative to the Central (15%) and NEA (17%) regions. These values are lower than those reported in the present study. Once again, these could be due to the use of different AIMs panels, with a reduced number of AIMs used to estimate ancestry in the study by Corach *et al* ((24 AIMs), and differences in the ascertainment of individuals in each study. The study by Corach *et al* included 246 male donors from paternity testing clinics.

In our study, we found the most significant differences in genetic ancestry proportions among individuals from NWA when compared to individuals from all other regions investigated. Currently, the Argentine northwest has approximately 5,000,000 inhabitants, who represent about 1/8 of the total country population. When the Spanish conquistadores arrived in the early 1500s, this region had the largest population size of the Argentine territory, with an estimated population of 200,000 [40]. Since the nineteenth century, with the increased development of agriculture, the central Pampas became the most developed region of Argentina, which consequently attracted many people to relocate there. Given the peripheral location of NWA region, and greater job opportunities in Buenos Aires City, few Europeans settled in NWA during the great flood of immigration that the country received the last decades of the nineteenth and early twentieth centuries. For these historical reasons, NWA is the region of Argentina where one would expect the largest Indigenous American ancestry component, and this is what we found among individuals ascertained from this region, from the province of Salta. Our results are consistent with those obtained by Wang *et al* [7] for individuals from the same province (Indigenous American ancestry of 65% and 72% for our study and Wang's, respectively). Interestingly, these authors reported variability within NWA, with more indigenous contribution in the north of the region (Salta province) than in the south (Tucuman provice). In our study we only ascertained individuals from the province of Salta so we were unable to investigate intra-regional variability in the NWA.

In the present study we were able to compare the distribution of genetic ancestry of the two northern regions of the country (NWA and NEA). We observed that individuals from NEA had a greater proportion of European ancestry compared to individuals from NWA. However, we also observed a smaller degree of variation of the Indigenous American and European components in NEA compared to NWA (NEA Standard Deviation (SD) = 0.13 and NWA SD = 0.24; variance ratio test p = 0.0014). This can be interpreted as the result of more widespread admixture in NEA. The historical data seems to support this assertion. Specifically, although the Spanish authorities tried to restrict inter-ethnic marriages and the use of indigenous languages across all regions of their colonies, the NEA represented a marginal area where this control was not very effective [41]. Current evidence suggestive of extensive social admixing is the common use of Guarani, a native language from the ancestral Amerindian tribes of this region, which is currently still in use by many people in this region independently of ancestral origin, even among those with little indigenous ancestry [41].

The Buenos Aires Metropolitan Area (Buenos Aires City and surrounding urban areas) is the third most populated metropolis in Latin America, after Mexico City and Sao Paulo. In Buenos Aires City two historical events had a strong influence in the genetic composition of its inhabitants. First, the arrival of a large number of European immigrants, mostly from Italy and Spain, between 1870 and 1950 who intermixed with the smaller local population Buenos Aires City. This was a population that had already resulted from admixture of several generations of original Indigenous Americans, Africans brought as slaves during the Spanish conquest in the 16^th^ and 17^th^ century, and the earlier Spanish conquistadores [42]. The second event was the influx of a second wave of immigration in the 1940s, when the industrial development of Buenos Aires City attracted people from other provinces and the bordering countries, who relocated to Buenos Aires City. These new immigrants had high Hispano-Amerindian genetic ancestry, and thus contributed this to the already admixed population of Buenos Aires City. This last group of migrants mostly settled in the 2^nd^ urban belt, where we observed significantly higher Indigenous American ancestry [4].

We validated our ancestry estimates with two approaches. One, by comparing ancestry estimates obtained with our panel of 99 AIMs to those obtained with 118,192 SNPs in a subset of individuals. Overall, we observed that the individual ancestry estimates that we obtained using information from 99 AIMs were strongly correlated with those obtained with genome wide data for the major ancestral components (European and Indigenous American). However, this was not the case for African ancestry, which shows a correlation coefficient of about 0.12. This low level of correlation is likely the result of an overestimation of the African component, as estimated by the 99 AIMs panel. Since genetic ancestry estimates have statistical variance, when the proportion of ancestry is close to zero the estimates of ancestry tend to be biased towards higher numbers (since the model does not allow for <0 ancestry). Therefore, care should be taken in interpreting ancestry estimates when the overall proportion of that ancestral group is low (<5%).

Another way of validating our results was to compare the obtained genealogical information to the estimated proportions of genetic ancestry, and to investigate how informative one would be of the other. We observed that the average estimated European ancestry among individuals with at least one European grandparent was higher than that of individuals with no European born grandparents (80% versus 54%). Therefore, our data showed that the number of grandparents born in Europe is highly correlated with the proportion of European ancestry as measured by genetic markers. Interestingly, our data indicated that the largest change in average genetic ancestry when considering the number of grandparents born in Europe was between 0 and 1 grandparent. This is probably reflecting the effect of assortative mating [18], suggesting that if one of the grandparents was born in Europe, it is likely that the person chosen as a partner would have been similar in terms of origin, and thus genetic ancestry. We can conclude that certain genealogical information, such as number of grandparents of European origin, could be a strong predictor of genetic ancestry in samples from Argentina. However, our results also suggest that caution should be taken when using genealogical information to predict genetic ancestry. Specifically, we observed that two individuals in the group of people with 4 grandparents from Europe had ∼34% of Indigenous American ancestry. This level of Indigenous American ancestry is likely to be the result of true Indigenous ascent rather than statistical noise in the genetic ancestry measurement; thus suggesting that misreporting of genealogical information is an important issue to consider. In this regard, we note that in Latin America in general, and Argentina in particular, European ancestry tends to be socially perceived as more “desirable” than Indigenous American ancestry. This may explain why historical sources and geographical atlases have usually estimated a greater European contribution compared to what has been estimated from various genetic studies [18]. The presence of relatively high Indigenous American ancestry among individuals in our study who reported four European grandparents suggests lack of knowledge about the ancestral origin of some family members.

Our study contrasted the similarities and differences in the ancestral composition of three Latin American cities with very different demographic histories: Mexico City, with a strong Indigenous American component, Buenos Aires City, with a strong European component, and San Juan de Puerto Rico, with a strong European as well as a relatively important African component. Despite the differences in the average genetic ancestry proportions between the three cities, our results show that the distributions of individual ancestry estimates have a similar degree of dispersion.

One limitation of the present study is that we ascertained individuals through a limited network of blood donor centers at hospitals and clinics, instead of using a population-based approach. Therefore, we cannot generalize the ancestry proportions obtained from each group of individuals to those in the region of origin of each group. A larger network of hospitals and clinics for our ascertainment, or a general population-based approach (e.g. random-digit dialing) would have given us a more precise picture of the distribution of ancestry proportions at the regional level. However, in spite of this limitation we were able to achieve our aims of describing the level of heterogeneity within the country as well as testing the reliability of genetic ancestry estimates using AIMs when comparing to grandparental origin.

In summary, our results suggest, in concordance with previous studies, that genetic epidemiological research in Latin America should take genetic ancestry into account, preferably by directly estimating it using AIMs or comparable genetic markers (e.g. GWAS data). Studies that are unable to obtain information about genetic ancestry should, at the minimum, take into consideration not only the countries and the regions of origin of all participating individuals, but also the cities from where individuals come from. As we report here, demographic variations at the local level could also affect admixture patterns, and thus confound associations. Self-reported information about grandparents' origins may be useful surrogates, especially in regions with recent immigration patterns. However this information has to be taken with extreme caution given the potential overestimation of European ancestry by self-report.

## Supporting Information

Figure S1 **Map of Federal District and first, second and third belt.**
(TIF)

Figure S2 **1^st^ and 2^nd^ Multidimensional components of samples from Buenos Aires metropolitan area and ancestral individuals (black triangles).** Samples from the Capital are represented in green, from the 1^st^ urban belt in pink, and from the 2^nd^ urban in orange.
(TIF)

Figure S3 **1^st^ and 2^nd^ Multidimensional components of samples from the cities of Buenos Aires, Mexico and San Juan, including European, African and Indigenous American ancestrals (black triangles).** Samples from Buenos Aires are in gold, samples from Mexico are in light blue and samples from Puerto Rico are in purple.
(TIF)

Table S1 **Individual information on the number of grandparents born in Argentina (by region), other South American country or Europe.**
(XLS)

Table S2 **List of ancestry informative markers (AIMs) with chromosomal position and allele frequency in European, African and Indigenous American samples.**
(XLSX)

Table S3 **Correlation coefficients between genome wide ancestry estimates and 99 ancestry informative markers (AIMs) estimates.**
(DOC)
