# Supplementary figures and images for: Heterogeneity in Genetic Admixture across Different Regions of Argentina
Source: PLoS One. 2012 Apr 10;7(4):e34695. doi: 10.1371/journal.pone.0034695 (PMC3323559; doi:10.1371/journal.pone.0034695)

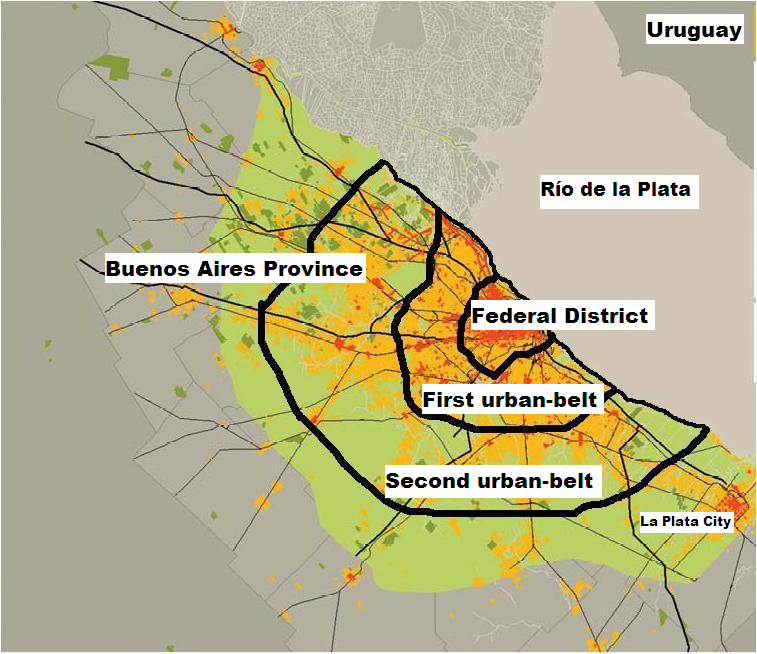

Supplement: Figure S1 — Map of Federal District and first, second and third belt. (TIF) [file pone.0034695.s001.tif]

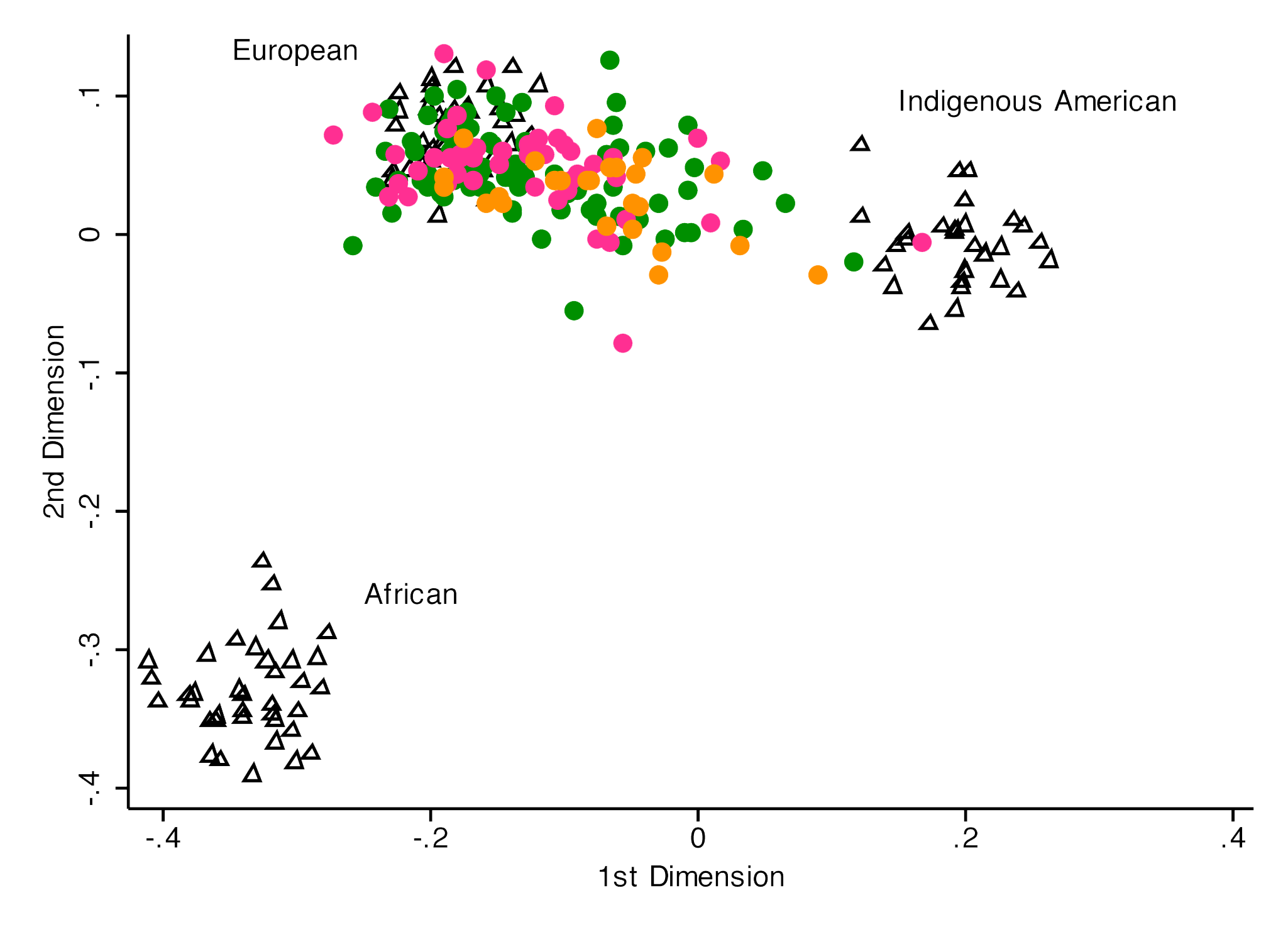

Supplement: Figure S2 — 1st and 2nd Multidimensional components of samples from Buenos Aires metropolitan area and ancestral individuals (black triangles). Samples from the Capital are represented in green, from the 1st urban belt in pink, and from the 2nd urban in orange. (TIF) [file pone.0034695.s002.tif]

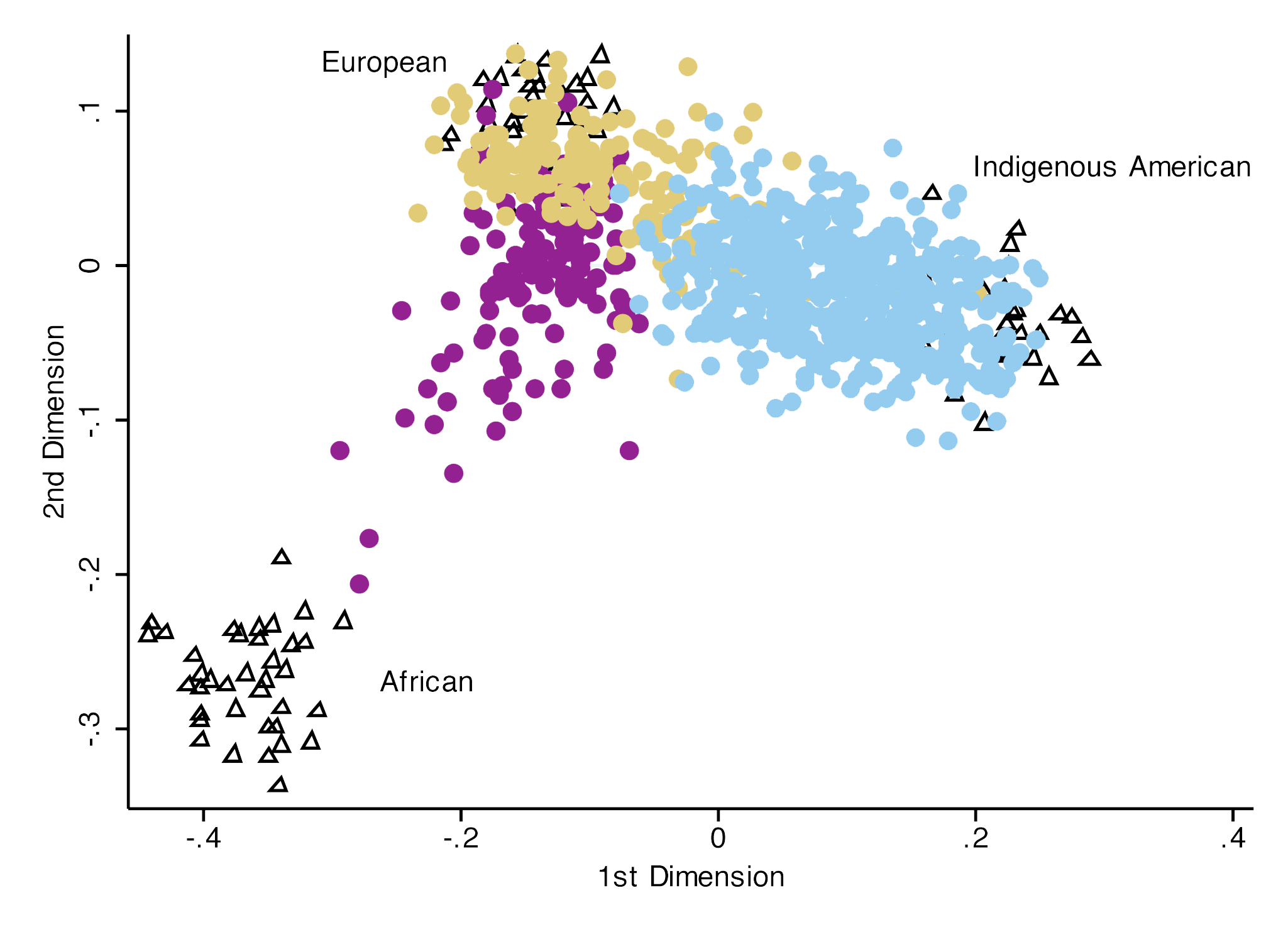

Supplement: Figure S3 — 1st and 2nd Multidimensional components of samples from the cities of Buenos Aires, Mexico and San Juan, including European, African and Indigenous American ancestrals (black triangles). Samples from Buenos Aires are in gold, samples from Mexico are in light blue and samples from Puerto Rico are in purple. (TIF) [file pone.0034695.s003.tif]
