# Supplementary material for: Heterogeneity in Genetic Admixture across Different Regions of Argentina
Source: PLoS One. 2012 Apr 10;7(4):e34695. doi: 10.1371/journal.pone.0034695 (PMC3323559; doi:10.1371/journal.pone.0034695)
Supplement: Table S3 — Correlation coefficients between genome wide ancestry estimates and 99 ancestry informative markers (AIMs) estimates. (DOC) [file pone.0034695.s006.doc]

Supplementary Material Table 1: Correlation coefficients between genome wide ancestry estimates and 99 AIMs estimates

| European | Admixture  (118,000 SNPs) | Frappe  (99 AIMs) | Structure  (99 AIMs) |
| --- | --- | --- | --- |
| Admixture (118,000 SNPs) | 1.00 |  |  |
| Frappe (99 AIMs) | 0.91 | 1.00 |  |
| Structure (99 AIMs) | 0.91 | 0.99 | 1.00 |
| Maximum Likelihood (99 AIMs) | 0.90 | 0.98 | 0.98 |
| Indigenous American |  |  |  |
| Admixture (118,000 SNPs) | 1.00 |  |  |
| Frappe (99 AIMs) | 0.94 | 1.00 |  |
| Structure (99 AIMs) | 0.93 | 1.00 | 1.00 |
| Maximum Likelihood (99 AIMs) | 0.93 | 0.99 | 1.00 |
| African |  |  |  |
| Admixture (118,000 SNPs) | 1.00 |  |  |
| Frappe (99 AIMs) | 0.11 | 1.00 |  |
| Structure (99 AIMs) | 0.12 | 0.96 | 1.00 |
| Maximum Likelihood (99 AIMs) | 0.12 | 0.85 | 0.82 |
